# Supplementary material for: Causal Relationship between Aging and Anorexia Nervosa: A White-Matter-Microstructure-Mediated Mendelian Randomization Analysis
Source: Biomedicines. 2024 Aug 16;12(8):1874. doi: 10.3390/biomedicines12081874 (PMC11351342; doi:10.3390/biomedicines12081874)
Supplement: Supplementary file 1 [file biomedicines-12-01874-s001.zip › Supplementary Figure/Figure S4/Singe SNP plot of MR analysis for TL to ubm-b-2038.pdf]

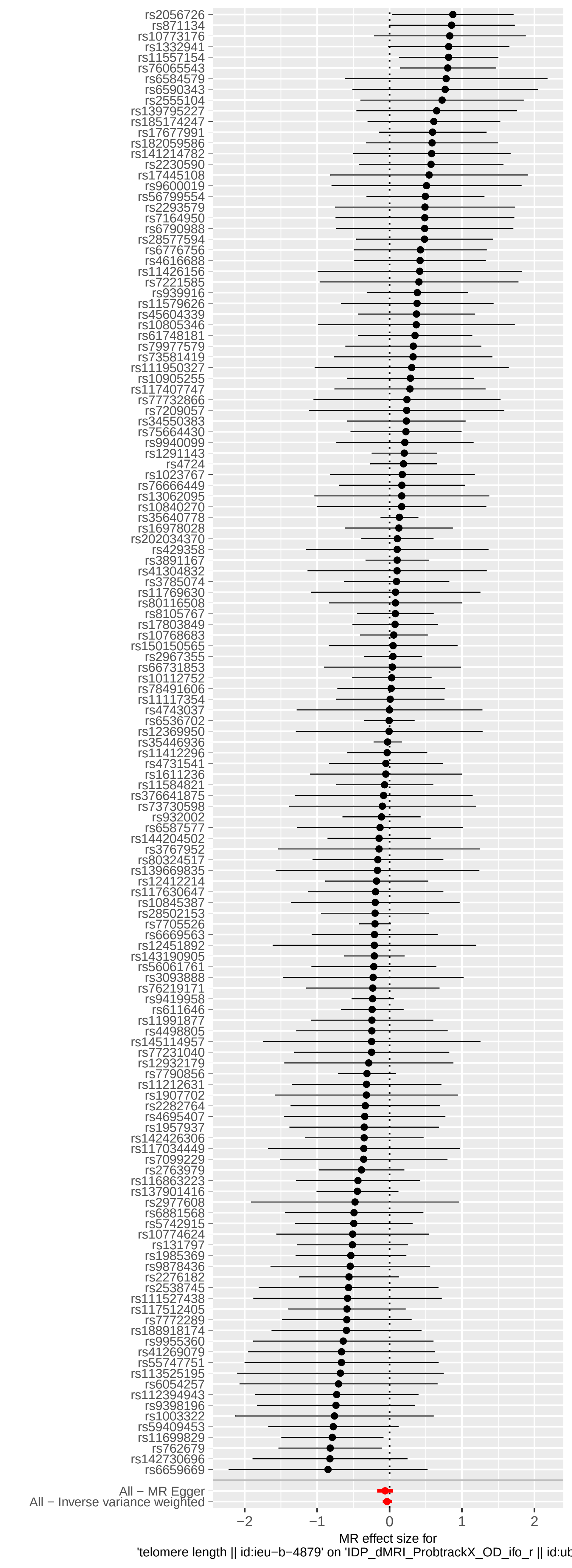
rs2056726
rs871134
rs10773176
rs1332941
rs11557154
rs76065543
rs6584579
rs6590343
rs2555104
rs139795227
rs185174247
rs17677991
rs182059586
rs141214782
rs2230590
rs17445108
rs9600019
rs56799554
rs2293579
rs7164950
rs6790988
rs28577594
rs6776756
rs4616688
rs11426156
rs7221585
rs939916
rs11579626
rs45604339
rs10805346
rs61748181
rs79977579
rs73581419
rs111950327
rs10905255
rs117407747
rs77732866
rs7209057
rs34550383
rs75664430
rs9940099
rs1291143
rs4724
rs1023767
rs76666449
rs13062095
rs10840270
rs35640778
rs16978028
rs202034370
rs429358
rs3891167
rs41304832
rs3785074
rs11769630
rs80116508
rs8105767
rs17803849
rs10768683
rs150150565
rs2967355
rs66731853
rs10112752
rs78491606
rs11117354
rs4743037
rs6536702
rs12369950
rs35446936
rs11412296
rs4731541
rs1611236
rs11584821
rs376641875
rs73730598
rs932002
rs6587577
rs144204502
rs3767952
rs80324517
rs139669835
rs12412214
rs117630647
rs10845387
rs28502153
rs7705526
rs6669563
rs12451892
rs143190905
rs56061761
rs3093888
rs76219171
rs9419958
rs611646
rs11991877
rs4498805
rs145114957
rs77231040
rs12932179
rs7790856
rs11212631
rs1907702
rs2282764
rs4695407
rs1957937
rs142426306
rs117034449
rs7099229
rs2763979
rs116863223
rs137901416
rs2977608
rs6881568
rs5742915
rs10774624
rs131797
rs1985369
rs9878436
rs2276182
rs2538745
rs111527438
rs117512405
rs7772289
rs188918174
rs9955360
rs41269079
rs55747751
rs113525195
rs6054257
rs112394943
rs9398196
rs1003322
rs59409453
rs11699829
rs762679
rs142730696
rs6659669
All − MR Egger
All − Inverse variance weighted
−2
−1
0
1
2
MR effect size for
'telomere length || id:ieu−b−4879' on 'IDP_dMRI_ProbtrackX_OD_ifo_r || id:ub
